# Application of Diethylzinc/Propyl Gallate Catalytic System for Ring-Opening Copolymerization of *rac*-Lactide and ε-Caprolactone

**DOI:** 10.3390/molecules24224168

**Published:** 2019-11-17

**Authors:** Rafał Wyrębiak, Ewa Oledzka, Ramona Figat, Marcin Sobczak

**Affiliations:** 1 Department of Biomaterials Chemistry, Chair of Analytical Chemistry and Biomaterials, Faculty of Pharmacy, Medical University of Warsaw, 1 Banacha St., Warsaw 02-097, Poland; rafal.wyrebiak@wum.edu.pl (R.W.); eoledzka@wum.edu.pl (E.O.); 2 Department of Environmental Health Sciences, Faculty of Pharmacy, Medical University of Warsaw, 1 Banacha St., Warsaw 02-097, Poland; ramona.figat@wum.edu.pl

**Keywords:** ring-opening polymerization, zinc catalyst, ε-caprolactone, rac-lactide, biodegradable polyesters, biomedical polymers

## Abstract

Biodegradable polyesters gain significant attention because of their wide potential biomedical applications. The ring-opening polymerization method is widely used to obtain such polymers, due to high yields and advantageous properties of the obtained material. The preparation of new, effective, and bio-safe catalytic systems for the synthesis of biomedical polymers is one of the main directions of the research in modern medical chemistry. The new diethylzinc/propyl gallate catalytic system was first used in the copolymerization of ε-caprolactone and rac-lactide. In this paper, the activity of the new zinc-based catalytic system in the copolymerization of cyclic esters depending on the reaction conditions was described. The microstructure analysis of the obtained copolyesters and their toxicity studies were performed. Resulted copolyesters were characterized by low toxicity, moderate dispersity (1.19–1.71), varying randomness degree (0.18–0.83), and average molar mass (5300–9800 Da).

## 1. Introduction

In recent years, biodegradable and bioresorbable homo- and copolyesters based on cyclic monomers: rac-lactide (rac-LA), L,L-lactide (LLA), ε-caprolactone (CL), glycolide (GL), and trimethylene carbonate (TMC) were widely tested for their potential use in biomedical applications [1,2]. These applications include sutures, drug delivery systems (e.g., drug nano- or microcarriers or macromolecular prodrugs), implants [3] and tissue engineering [4].

Although Poly(ε-caprolactone) (PCL) exhibits favorable biocompatibility and mechanical properties, it biodegrades in vivo very slowly—from a few months to several years [5]. Polylactide (PLA) displays variable biodegradation time, ranging from several weeks to about two years [6]. The exact biodegradation rate of PLA depends on the polymer’s average molecular weight and its dispersity (Đ), crystallinity, microstructure, etc. Co- or terpolymers of CL, rac-LA, LLA, GL, or TMC characterized by various microstructures allow obtaining the assumed time of polymer biodegradation and high controlled release of active substances from the polymeric carrier [7].

Biomedical polyesters can be mainly obtained by two methods: ring-opening polymerization (ROP) or polycondensation. The ROP process exhibits numerous advantages over classic polycondensation, such as higher product yield, proper average molecular weight, and its Đ [8]. Most of the biomedical polyesters are synthesized in the presence of the tin (II) 2-ethylhexanoate (SnOct_2_) initiator, which was approved by the United States Food and Drugs Administration (FDA) as a food additive [5]. However, tin derivatives are virtually irremovable from the final biomaterial. This may present a serious health risk due to their toxicity, especially toward juveniles [9,10,11,12]. Furthermore, possible, industrial application of large quantities of this compound also raises environmental concerns [13]. Several alternative ROP initiators/catalysts for the polyester synthesis were investigated over the years, such as ionic [14], coordinating [15], and enzymatic [16]. A very interesting group of coordinating initiators/catalysts are zinc [17], calcium [9] and zirconium (IV) acetylacetonates [18], which are characterized by high activity and biocompatibility. Some of the other examples of ROP catalysts are group 3 metal complexes [19,20], bismuth (III) analogs of SnOct_2_ [21], organic complexes of aluminum [22,23], alkoxyl titanium [24], or alkyl/alkoxyl tin compounds [25]. Metal-organic ROP catalysts seem to be the most favorable due to their selectivity. They allow obtaining—depending on the reaction conditions—polymeric products with variable microstructure. Since the microstructure of the polymer determines its hydrolytic stability, it is possible to synthesize polyesters with varying biodegradation rates by modifying polymerization conditions, such as reaction time, temperature, or used catalyst [7,26].

Zinc salts, such as lactate [13], aceturate, or L-prolinate [27] were found to be active catalysts for ROP of LLA. Organic zinc complexes were also successfully used as initiators/catalysts for the homopolymerization of LA [28,29,30,31], CL [32,33] or copolymerization of CL, and LA [34,35,36]. Zinc-based catalysts may be a viable alternative to SnOct_2_ due to low toxicity and programmable polymer chain microstructure. As our previous studies have shown [37,38,39], diethylzinc/propyl gallate (ZnEt_2_/PGA) was an efficient catalytic system for the homopolymerization of rac-LA and CL, yielding polymers with varying microstructure (depending on the reaction conditions). It is worth mentioning that propyl gallate (PGA) is used as an additive (E310) in pharmaceuticals, cosmetics, and food due to its antioxidant properties [40]. Most importantly, resulting polymers were found to be non-toxic [39]. Consequently, in this paper, the results of copolymerization of rac-LA and CL in the presence of these efficient and non-toxic ZnEt_2_/PGA catalytic systems have been presented.

## 2. Results and Discussion

**Figure 1:**
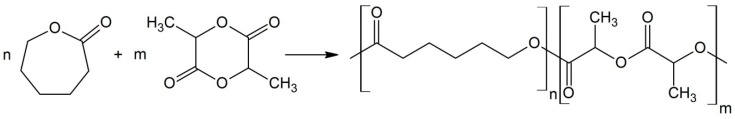
The scheme of copolymerization of rac-LA and CL.

The copolymerization of rac-LA and CL (Figure 1) was carried out for different monomer molar ratios, time, and temperature. The catalytic system was synthesized by the reaction of PGA with 3 molar equivalent of ZnEt_2_. PGA has been used as a bio-safe co-initiator of the ROP process. However, it is worth to mention that we were unable to establish the exact structure of the applied catalytic system. The attempts of its crystallization with numerous solvents led to the precipitating of the amorphous solid. Based on the reaction stoichiometry, however, we assume that the present phenolic O-Zn-Et groups may act as ROP initiators.

In our previous studies, gallic acid (GA) and PGA were used as co-initiators of the homopolymerization of rac-LA [38] and CL [37]. In those studies, toluene, tetrahydrofuran, or dichloromethane were investigated as a reaction medium. It was found that GA exhibited a tendency to promote macrolactonization during the ROP process. In addition, toluene has proved to be the optimal reaction medium of the ROP process [38]. As in vitro degradation of rac-LA is much faster than LLA [41], the former monomer was used to reduce polymer degradation time. Considering these facts, in current work, we decided to use ZnEt_2_/PGA catalytic system and toluene as a reaction medium in the copolymerization process of rac-LA and CL. 

The effect of the temperature and reaction time on the yield of the product, the products average molecular weight, as well as microstructure of the synthesized materials were investigated (Table 1).

### 2.1. Polymers Characterization

It is well known that the polyester microstructure influences the rate of the polymer biodegradation and kinetics of drug release. The microstructure of the polymer chain depends on the intra- or intermolecular transesterification and stereoselectivity process. Transesterification is a well-known phenomenon causing polyester sequence redistribution. In short, during the ROP of LA, lactyl units (L) should appear in pairs in the final polymer chain. However, transesterification may lead to the appearance of abnormal sequences, defined as type-II transesterification [42].

The microstructure of the obtained copolymers was established by ^13^C NMR studies. Type-II transesterification ratios, average lengths of monomer blocks, and randomness degrees were measured by NMR signal area calculations [43,44].

As is shown in Table 1, the yield of the ROP process depended on the monomer/catalytic system’s ratio, temperature, and reaction time.

In most cases, the CL conversion was faster and higher than that of LA. The extension of the reaction time up to 16 h at 80 °C did not significantly affect it. However, it was necessary to carry out polymerization for up to 48 h at 60 °C in order to achieve satisfying conversion. The average length of lactydyl units was decreased with increasing of the reaction time, that indicates the occurrence of transesterification.

Increasing of the ROP temperature generally increased the product yield. Interestingly, it seemed that the combination of a high catalyst concentration (6/100 and 8/100), high temperature (80 °C), and long reaction time (24 and 48 h) negatively affected the reaction yield. It is possible that a high concentration of the active centers on the catalyst led to the degradation of already formed polyester chains. Oligomers could have been removed from the final product during the purification step (during precipitation from cold methanol).

Generally, *M*_n_ increased with the increasing of the reaction time. A positive correlation between *M*_n_ and the reaction temperature was also observed. *Đ* was found to increase with increasing the reaction time, temperature, and lactydyl unit concentration in the obtained polyester.

**Figure 2:**
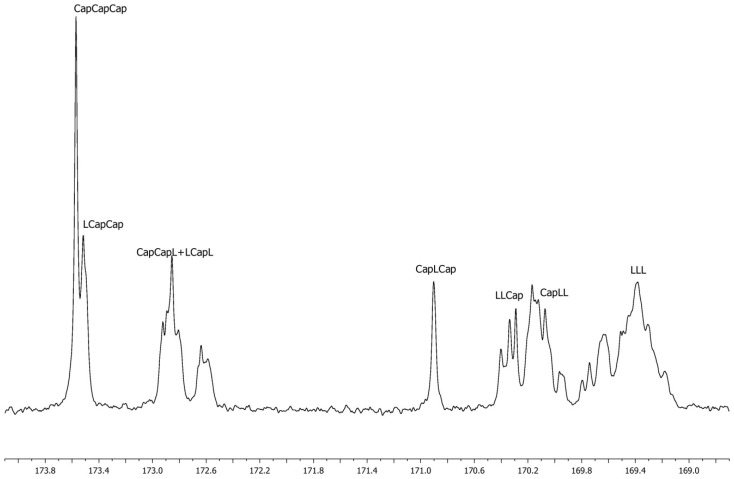
Typical ^13^C NMR spectrum of the obtained copolymers (carbonyl region).

Type-II transesterification was present in most obtained copolymers, as a signal from the CapLCap triad (lactyl-caproyl-lactyl units) around 170.8 ppm with varying intensity was observed (see Figure 2). The occurrence of this phenomenon is unsurprising, as Zn initiators tend to promote it in higher temperatures [42]. The Zn/monomer feed ratio affected the mentioned parameter, namely 2/100 ratio was highly favored; 1/100 ratio was favored moderately, while 8/100 and 6/100 ratios was inhibited. As was expected, the increase in the polymerization temperature and reaction time increased the T_II_ ratio. Some occurring deviations might have resulted from the ^13^C NMR measurements low accuracy. The degree of randomness increased (or supposedly reached plateau) with reaction time increase. A high catalyst load seemed to reduce this parameter.

Reactivity ratios: r_1_ (M_1_ = rac-LA) and r_2_ (M_2_ = CL) were roughly estimated based on the monomer feed in the reaction and composition of the resulted copolymers [41,45,46]. It was found that r_1_ was higher than r_2_; these values were in the range of 3.13–20.6 and 1.12–11.2, respectively. Both r_1_ and r_2_ were higher than 1, which indicated non-azeotropic, non-ideal copolymerization.

In summary, the 2/100 Zn/monomer feed ratio was found to be unfavorable due to its tendency to promote exceptionally high T_II_: from 39% for entry 17 up to 100% for entry 18. The 6/100 and 8/100 monomer feed ratios were advantageous as a satisfactory compromise between high yield and low T_II_. The 60 °C reaction temperature was beneficial considering low T_II_, but also negatively affected yield, e.g., 20% yield for entry 19 and 25% yield for entry 24. However, this effect can be largely negated by extending the reaction time to 48 h: 46% yield for entry 20, 52% yield for entry 25, and 46% yield for entry 30. In connection with the above, the optimal reaction time is 16 h (48 h for the reactions performed at 60 °C). When the ROP process has been carried out in longer reaction time, T_II_ and Đ values were higher.

Considering optimal reaction conditions, entries 21 (CL/rac-LA molar ratio 1/1, Zn/monomers molar ratio 6/100, T = 80 °C, reaction time: 16 h, 81% yield, M_n_ = 8400 Da, Đ = 1.38, T_II_ = 6.5%), 26 (CL/rac-LA molar ratio 2/1, Zn/monomers molar ratio 8/100, T = 80°C, reaction time: 16 h, 83% yield, M_n_ = 8600 Da, Đ = 1.38, T_II_ = 6.1%) and 31 (CL/rac-LA molar ratio 1/2, Zn/monomers molar ratio 8/100, T = 80°C, reaction time: 16 h, 73% yield, M_n_ = 8000 Da, Đ = 1.54, T_II_ = 3.0%) were favorable, as they combined high products yield and relatively low T_II_ data.

**Figure 3:**
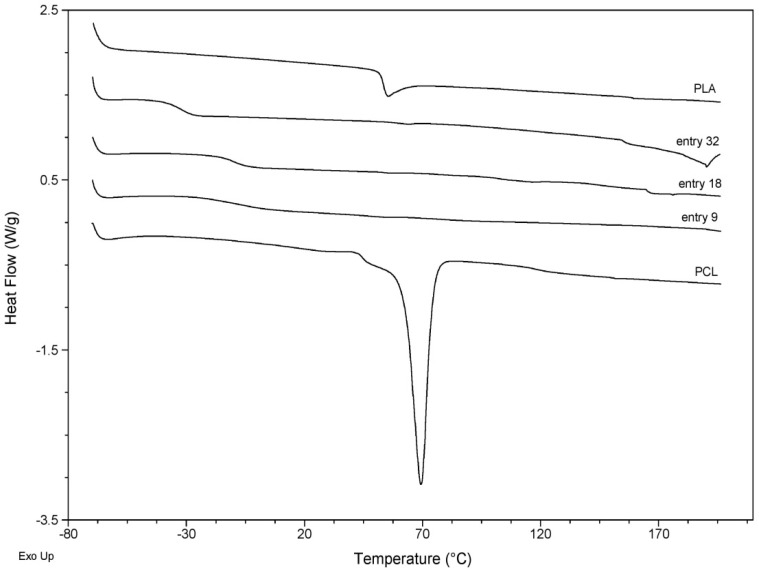
DSC curves of the selected samples and references.

The thermal properties of the obtained copolymers were also investigated by DSC. The results are shown in Figure 3. The melting temperature (T_m_) and glass transition temperature (T_g_) of the selected copolymers and references were determined and listed in Table 2. PCL as the reference was showing T_m_ = 69.4 °C and T_g_ = −60.0, whereas for PLA T_g_ = 53.4 °C. As is evidenced in Figure 3, T_m_ was not detected for PLA as the reference. The T_m_ value for the synthesized copolymers entitled as entry 9 (Figure 3) was detected at 51.6 °C. However, for the synthesized samples, the entitled as entries 18 and 32, next to the T_m_ values of 52.5 °C and 56.4 °C, respectively, extended peaks have emerged, centered as 164.9 and 181.8 °C, that are characteristic for poly(d,l-lactide) (PDLA) [47]. 

Significant changes in T_g_ values were, however, observed for the synthesized copolymers compared to the references (Table 2). They were −5.32 °C, −9.74 °C, and −32.4 °C for entries 9, 18, and 32, respectively. Higher T_g_ is generally a favorable property since compounds with high T_g_ have a reduced ability to recrystallize at a given temperature, compared to those that have a lower T_g_. These results were attributed to the copolymerization process, which decreases the mobility of copolymer chains, a fact supported in the literature [48].

### 2.2. Toxicity Studies

In the Spirotox test, it was found that none of the tested samples had been toxic to the protozoan *S*. *Ambiguum*. Some of the samples exhibited low toxicity toward luminescent bacteria *Allivibrio Fischeri* (The percent of a toxic effect (PE) from 21 to 34, Table 3). However, regarding the standard deviation, only entries 5 and 9 could be unambiguously considered slightly toxic. The toxic effect of these samples could have been caused by insufficient removal of catalytic system traces from the final polymer. In the umu-test with *S*. *Typhimurium*, all of the tested samples showed no cyto- and genotoxic potential with or without metabolic activation (IR < 1.5, Table 4).

## 3. Materials and Methods 

### 3.1. Materials

All chemicals were stored in an inert atmosphere of dry argon. Chloroform-d for NMR (99.8 atom % D, with 0.1 *v*/*v*% TMS, stabilized with silver), and toluene (99.85%, Extra Dry, over Molecular Sieves, AcroSeal®) were purchased from Acros Organics (Geel, Belgium) and used as received. Rac-LA (rac-lactide, 3,6-dimethyl-1,4-dioxane-2,5-dione), ZnEt_2_ solution (15% diethylzinc in toluene), PGA (propyl gallate, 98%+), poly(d,l-lactide) (viscosity 0.68 dL/g), and polycaprolactone (average M_w_ ca. 14,000; average M_n_ ca. 10,000) were purchased from Sigma-Aldrich Co. (Poznan, Poland) and used as received. CL (ε-caprolactone, 6-caprolactone, 99%+) was purchased from Sigma-Aldrich Co. (Poznan, Poland) and stored over 5 A° molecular sieves. Dichloromethane (pure, 99%) and methanol (pure, 99.9%) were purchased from Chempur (Piekary Śląskie, Poland) and distilled before use.

### 3.2. Methods

#### 3.2.1. Synthesis of the Catalytic System

The catalytic system was prepared using air-free techniques. PGA (128 mg, 0.603 mmol) and magnetic stir bar were placed in a 25 mL one-necked round-bottom flask. The high vacuum was applied for 1 h. After that, the flask was evacuated and backfilled with argon three times, and 8.37 mL of dry toluene was added. The flask was sealed, and the reaction was stirred overnight. Next, the reaction mixture was cooled to 0 °C, and 1.63 mL of 15% ZnEt_2_ (1.81 mmol, three equivalents) solution was added. The reaction was stirred further for 2 h with cooling, after that, the mixture was allowed to warm to room temperature. The prepared solution contains ca. 0.18 mmol of Zn in 1 mL.

#### 3.2.2. Polymerization Procedure

Copolymerization was carried out in a vacuum-dried 10 mL glass tubes with the joint. A mixture of monomers in appropriate ratios (18 mmol total) was placed in a reaction vessel, which was evacuated and backfilled with argon three times. After that, 5 ml of dry toluene and 1 mL of the catalytic system (0.18 mmol of Zn) was added. Glass tube was sealed, shaken, placed in the preheated oil bath with thermostat and kept at the appropriate temperature and for the required amount of time. Then, the reaction mixture was removed from the oil bath, allowed to cool to room temperature, dissolved in dichloromethane, and washed two times with 5% HCl solution and one time with distilled water. The product was precipitated by adding a concentrated dichloromethane solution of polymer to cold (2–8 °C) methanol (ca. 10 times volume of dichloromethane solution). The solvent mixture was decanted, the precipitate was washed with cold methanol, dried in the air overnight, and in vacuum for ca. 48 h.

#### 3.2.3. Measurements

The polymerization products were characterized in a deuterium chloroform solution by means of ^1^H- (300 MHz) and ^13^C-NMR (75 MHz) spectroscopy (Varian, LabX, Midland, ON, Canada).

Relative average molecular mass and molecular mass distribution were determined by Gel permeation chromatography (GPC). GPC instrument (GPC Max + TDA 305, Viscotek, Malvern, UK) was equipped with Jordi DVB Mixed Bed columns (one guard and two analytical) at 30 °C in CH_2_Cl_2_ (HPLC grade, Sigma-Aldrich, Poznań, Poland), at a flow rate of 1 mL/min with RI detection and calibration based on narrow PS standards (ReadyCal Set, Fluka, Poznan, Poland). The results were processed with OmniSEC software (version 4.7). *M*_n_ values were not corrected.

A differential scanning calorimetry technique (DSC, TA Instruments, New Castle, USA) was used to analyze the thermal transitions of the polymers. The DSC data were obtained between −80 and 200 °C using the Q200 apparatus. The sample was heated and cooled at a rate of 10 °C min^−1^. An empty T_zero_ aluminum pan was used as the reference. 

#### 3.2.4. Spectroscopic Data

Typical NMR shifts ranges were as follows: ^1^H NMR δ 5.19–5.10 (m, C(O)C**H**(CH_3_)O), 4.11 (br s, CH_2_C**H**_2_OC(O)CH(CH_3_)), 4.04 (t, CH_2_C**H**_2_OC(O)CH_2_CH_2_), 2.37 (br s, CH(CH_3_)OC(O)C**H**_2_CH_2_CH_2_), 2.28 (t, CH_2_CH_2_OC(O)C**H**_2_CH_2_CH_2_), 1.64–1.36 (br m, CH(C**H**_3_), C(O)CH_2_C**H**_2_C**H**_2_C**H**_2_CH_2_O) ppm; ^13^C NMR δ *ca.* 172.8–172.0 (**C**(O)CH_2_CH_2_CH_2_CH_2_CH_2_O), 171.0–170.8 (CH_2_CH_2_O**C**(O)CH(CH_3_)OC(O)CH_2_CH_2_), 170.3–168.7 (O**C**(O)CH(CH_3_)O), 69.3–67.8 (OC(O)**C**H(CH_3_)O), 64.8–64.4 (C(O)CH_2_CH_2_CH_2_CH_2_**C**H_2_O), 33.5–32.9 (C(O)CH_2_CH_2_CH_2_CH_2_CH_2_O), 27.9–27.5 (C(O)CH_2_CH_2_CH_2_**C**H_2_CH_2_O), 25.0–24.5 (C(O)CH_2_CH_2_**C**H_2_CH_2_CH_2_O), 24.2–23.7 (C(O)CH_2_**C**H_2_CH_2_CH_2_CH_2_O), 16.7–16.3 (OC(O)CH(**C**H_3_)O) ppm.

### 3.3. Toxicity Studies

#### 3.3.1. Microtox and Spirotox Tests

Five milligrams of the copolymer was placed in the glass tube with 5 mL of Tyrode’s solution (Spirotox) or 2% NaCl (Microtox). The tubes were incubated at 37 °C for 24 h with shaking.

Microtox: A short-term bioassay with the luminescent bacteria *Allivibrio fischeri* (previously known as *Vibrio fischeri*). The procedure was based on the International Organization for Standardization (ISO) standard [49]. Shortly, the tested and the control (2% NaCl) samples were incubated with the bacteria at 15 °C for 15 min, and the luminescence was measured in the Microtox M500 luminometer. Then the percent of inhibition of the luminescence was calculated in comparison to the control. 

Spirotox: A short-term bioassay with the ciliated protozoan *Spirostomum ambiguum*. The test was performed according to the ISO 11348-3:2007 standard protocol [50]. Shortly, the tested and the control (Tyrod solution) samples were incubated with the protozoans at 25 °C for 24 h, and the sublethal (deformations) and lethal effects are observed with the dissection microscope (magnification 10×). Then the percent of affected protozoans was calculated for the sample in comparison to the control.

#### 3.3.2. Umu—Test

*Umu*—a test that detects the induction of the SOS system in the strain *S. typhimurium* TA1535/pSK1002. SOS system is the bacterial response to the DNA-damaging agents. The test strain is genetically modified—the umuC gene activity is linked to the synthesis of β-galactosidase, while other DNA regions responsible for this enzyme synthesis were deleted. Therefore β-galactosidase activity strictly depends on the SOS system induction level and the genotoxic activity of the tested compound [51]. The enzyme converts colorless substrate (ortho-nitrophenyl-β-galactoside) into the yellow product, which can be quantified colorimetrically at 420 nm. Additionally, the bacteria growth (G) is evaluated by measurement of an optical density to determine the cytotoxicity of tested samples. The genotoxic potential of the sample is presented as the Induction Ratio (IR)—the β-galactosidase activity ratio of the tested sample in comparison to the negative control. Samples with IR ≥ 1.5 are considered as genotoxic. 

In the present study, the *umu*-test was carried out in the micro-plate variant according to the ISO 13829 guideline, with and without metabolic activation (S9 liver fraction from male Sprague-Dawley rats treated five days before the isolation with a single dose of 500 mg/kg body weight of Aroclor 1254 in soya oil) [49]. Deionized sterile water was used as a negative control, 2-aminoanthracene and 4-nitroquinoline N-oxide were used as positive controls, and phosphate-buffered saline (PBS from Gibco, Thermo Fisher Scientific, Darmstadt, Germany) as solvent control. All tested samples were incubated in PBS - 1 mg/mL for 24 h, 37 °C, with shaking. Before the assay, all extracts were sterilized by filtration (0.20 µm). All samples were tested in two-fold dilution series (four concentrations, the highest concentration of 0.66 mg/mL). 

## 4. Conclusions

In summary, the developed, non-toxic catalytic system enables the synthesis of rac-LA/CL copolymers. The obtained products were characterized by a wide range of *M*_n_ (from 5400 to 9800 Da), adequate for biomedical applications. It is worth to note that the copolyesters were found to be no cyto- nor genotoxic, and thus, they can be used in the drug formulation technology. Depending on the reaction conditions (monomer feed ratio, reaction temperature and time, catalyst feed), the produced copolyesters vary in their microstructure, thus can be used to obtain various drug delivery systems (middle- and short term), characterized by different drug release kinetics. We have also found that the optimal conditions for the copolymerization process were: 6/100 or 8/100 Zn/monomer ratio, 16 h reaction time, and 80°C reaction temperature. The copolymeric products obtained under these conditions were characterized by a high reaction yield and low type-II transesterification ratio.

## Figures and Tables

**Table 1:** Copolymerization conditions of CL and rac-LA.

Entry	Molar Ratio CL/*rac*-LA	Molar Ratio Zn/Monomers	Reaction Time [h]	Temp. [°C]	Conv.LA	Conv.CL	[L] ^a^	Yield ^b^ [%]	l_Cap_^c^	l_LL_^d^	R ^e^	*M*_n_^f^	*Đ* ^f^	T_II_ [%]
1	1/1	1/100	16	80	0.88	0.92	0.77	64	2.32	4.83	0.45	9000	1.42	31
2	1/1	1/100	24	80	0.85	0.96	0.60	83	2.24	2.65	0.47	9700	1.54	47
3	1/1	1/100	48	80	0.82	0.97	0.58	87	1.85	1.63	0.74	9000	1.61	74
4	2/1	1/100	16	80	0.76	0.96	0.50	65	3.13	1.82	0.55	9200	1.35	39
5	2/1	1/100	24	80	0.74	0.97	0.39	93	2.58	1.09	0.76	8000	1.46	67
6	2/1	1/100	48	80	0.75	0.97	0.43	85	3.03	1.34	0.65	9200	1.53	60
7	1/2	1/100	16	80	0.89	0.92	0.77	68	1.76	4.45	0.49	8600	1.60	38
8	1/2	1/100	24	80	0.85	0.93	0.68	90	1.68	3.34	0.47	7900	1.51	40
9	1/2	1/100	48	80	0.86	0.94	0.73	85	1.78	3.82	0.49	8600	1.70	23
10	1/1	2/100	16	80	0.80	0.92	0.65	64	2.00	2.16	0.67	5400	1.32	60
11	1/1	2/100	24	80	0.80	0.95	0.58	58	2.32	1.87	0.64	5400	1.24	40
12	1/1	2/100	48	80	0.88	0.92	0.58	63	2.41	1.82	0.66	5300	1.38	45
13	2/1	2/100	16	80	0.67	0.96	0.42	67	2.21	1.09	0.80	6000	1.39	89
14	2/1	2/100	24	80	0.73	0.96	0.46	69	2.61	1.18	0.79	6700	1.35	69
15	2/1	2/100	48	80	0.76	0.98	0.44	62	2.84	1.24	0.72	6700	1.45	60
16	1/2	2/100	16	80	0.88	0.92	0.78	67	1.70	3.96	0.58	7100	1.52	46
17	1/2	2/100	24	80	0.82	0.95	0.79	64	1.85	4.14	0.56	7100	1.42	39
18	1/2	2/100	48	80	0.84	0.87	0.77	66	1.27	2.67	0.83	7700	1.56	100
19	1/1	6/100	24	60	0.61	0.75	0.51	20	11.68	3.10	0.33	7700	1.22	7.4
20	1/1	6/100	48	60	0.90	0.83	0.64	46	5.19	7.51	0.18	8400	1.29	11
21	1/1	6/100	16	80	0.50	0.55	0.70	81	7.15	7.43	0.23	8400	1.38	6.5
22	1/1	6/100	24	80	0.55	0.65	0.72	79	10.07	2.54	0.69	8500	1.43	12
23	1/1	6/100	48	80	0.89	0.86	0.62	52	4.40	3.29	0.40	9600	1.51	28
24	2/1	8/100	24	60	0.83	0.29	0.79	25	8.12	12.94	0.19	7900	1.19	1.7
25	2/1	8/100	48	60	0.86	0.96	0.48	52	9.89	3.54	0.27	8400	1.26	9.6
26	2/1	8/100	16	80	0.78	0.93	0.49	83	7.76	4.05	0.24	8600	1.38	6.1
27	2/1	8/100	24	80	0.85	0.96	0.49	59	7.23	3.21	0.31	9200	1.36	14
28	2/1	8/100	48	80	0.84	0.96	0.49	32	8.82	2.95	0.33	9800	1.40	5.3
29	1/2	8/100	24	60	0.85	0.60	0.82	44	5.96	22.04	0.13	7300	1.32	0
30	1/2	8/100	48	60	0.90	0.87	0.80	46	6.01	15.96	0.16	8000	1.40	15
31	1/2	8/100	16	80	0.77	0.90	0.81	73	6.39	15.97	0.17	8000	1.54	3.0
32	1/2	8/100	24	80	0.88	0.90	0.80	42	3.97	13.31	0.19	8600	1.59	39
33	1/2	8/100	48	80	0.87	0.90	0.75	34	4.67	7.31	0.28	9300	1.71	12

^a^ Molar fraction of lactyl units in the polymer (determined by ^1^H NMR); ^b^ Isolated yield; ^c^ Average length of caproyl blocks; ^d^ Average length of lactydyl blocks; ^e^ Randomness degree; ^f^ Determined by GPC.

**Table 2:** Differential scanning calorimetry (DSC) results of the selected copolymers and homopolymers as references.

Entry	T_m1_ [°C] ^a^	T_m2_ [°C]	T_g_ [°C] ^b^
PCL	69.4	-	−60.0
PLA	-	-	53.4
9	51.6	-	−3.8
18	52.5	164.9	−10.0
32	56.4	181.8	−32.5

^a^ Melting temperature; ^b^ Glass transition temperature.

**Table 3:** The cytotoxicity results of the synthesized copolymers.

Entry	Spirotox 24 h-PE ^1^	Microtox 15 min-PE ^1^
1	0	13 ± 12
2	0	25 ± 6
3	0	10 ± 6
4	0	16 ± 3
5	0	34 ± 3
6	0	18 ± 4
7	0	10 ± 6
8	0	21 ± 3
9	0	22 ± 2

^1^ Percent of toxic effect.

**Table 4:** The results of the umu-test for the highest concentrations of the tested extracts (0.66 mg/mL).

Entry	−S9 ^a^		+S9 ^b^	
	G ^c^ ± SD	IR ^d^ ± SD	G ^c^ ± SD	IR ^d^ ± SD
1	1.02 ± 0.02	0.86 ± 0.11	0.91 ± 0.02	1.00 ± 0.02
2	1.00 ± 0.01	0.77 ± 0.11	0.90 ± 0.04	1.00 ± 0.31
3	1.05 ± 0.03	0.73 ± 0.08	0.91 ± 0.13	1.01 ± 0.15
4	1.08 ± 0.02	0.77 ± 0.08	1.03 ± 0.19	0.79 ± 0.07
5	1.00 ± 0.06	0.75 ± 0.09	0.87 ± 0.05	1.06 ± 0.02
6	0.97 ± 0.07	0.90 ± 0.14	1.05 ± 0.15	0.81 ± 0.09
7	1.13 ± 0.05	0.85 ± 0.06	1.04 ± 0.06	1.02 ± 0.08
8	1.02 ± 0.13	0.88 ± 0.28	1.01 ± 0.16	0.75 ± 0.09
9	0.99 ± 0.02	0.84 ± 0.13	1.07 ± 0.10	0.77 ± 0.09
Negative Control	1.01 ± 0.09	0.91 ± 0.17	1.00 ± 0.10	0.99 ± 0.14
Solvent Control	0.92 ± 0.12	0.83 ± 0.12	0.93 ± 0.07	0.84 ± 0.07

^a^ Without metabolic activation, ^b^ With metabolic activation, ^c^ Growth, ^d^ Induction Ratio.
